# Delayed Extraction of Abscessed Teeth

**Published:** 1906-04

**Authors:** 


### DELAYED EXTRACTION OF ABSCESSED TEETH.

Another trouble which presents for treatment, and which is, I regret to say, in many cases the result of poor treatment, is often characteristic of the mal-practitioner. It is the treatment applied to abscessed teeth, where extraction has been delayed several days, after it has been determined that extraction is necessary.

I have heard men say that the extraction of an abscessed tooth, during that period when the face is swollen is a very dangerous procedure, and I dare say that it is not an uncommon practice even in this profession to advise postponement of the extraction until the tissue resumes a normal condition, or the swelling "goes down." I have never, however, been able to get a satisfactory explanation as to why the extraction should be delayed. This is inflammation again, and, as always, the removal of the cause should be the first step in its treatment. The pathological changes which have taken place in the tooth or surrounding tissues cause it to be a foreign body, and the system tries to expel it. The anatomical conditions which are associated with its position, however, prevent this from taking place, therefore, the tooth acts as an irritant. All the other stages of inflammation follow this step and we get destruction of the tissues. I will show you a case on the screen later where the destructive process involved the malar and maxillary bones, parts of which I removed to bring about a cure.

There is no more reason why an abscessed tooth should be retained in the jaw simply because the face is swollen, than there would be to allow a splinter of wood to remain in the hand, a piece of glass in the foot, or a piece of steel in the eye, or for a gangrenous appendix to be left undisturbed until the inflammatory symptoms had subsided before attempting to remove it, since the forms of pathology are identical.

The fact that some cases of tooth extraction result in the death of the individual does not mean that the extraction of the tooth was the cause; such misadventure is usually due to the negligence of the operator in overlooking the very important steps of mouth sterilization before and after extraction. I base this statement upon the clinical fact that most, if not all, deaths following tooth extraction are due to septic pneumonia, or oral sepsis.—*Items of Interest.*

